# Decoding of the neural representation of the visual RGB color model

**DOI:** 10.7717/peerj-cs.1376

**Published:** 2023-05-11

**Authors:** Yijia Wu, Yanjing Mao, Kaiqiang Feng, Donglai Wei, Liang Song

**Affiliations:** 1 Fudan University, Fudan University, ShangHai, YangPu, China; 2 Shanghai Key Research Laboratory, Shanghai Key Research Laboratory, ShangHai, PuDong, China

**Keywords:** Decoding, Visual, Color, Machine learning, EEG, RGB

## Abstract

RGB color is a basic visual feature. Here we use machine learning and visual evoked potential (VEP) of electroencephalogram (EEG) data to investigate the decoding features of the time courses and space location that extract it, and whether they depend on a common brain cortex channel. We show that RGB color information can be decoded from EEG data and, with the task-irrelevant paradigm, features can be decoded across fast changes in VEP stimuli. These results are consistent with the theory of both event-related potential (ERP) and P300 mechanisms. The latency on time course is shorter and more temporally precise for RGB color stimuli than P300, a result that does not depend on a task-relevant paradigm, suggesting that RGB color is an updating signal that separates visual events. Meanwhile, distribution features are evident for the brain cortex of EEG signal, providing a space correlate of RGB color in classification accuracy and channel location. Finally, space decoding of RGB color depends on the channel classification accuracy and location obtained through training and testing EEG data. The result is consistent with channel power value distribution discharged by both VEP and electrophysiological stimuli mechanisms.

## Introduction

The most basic element of color is RGB (Aloise et al., 2011). RGB is the code of color red, green, blue in three channels. It is the color systems that are widely used and the colors which all most human vision can perceive (Pfurtscheller et al., 2000). Although the visual system has a responsibility to the different RGB color stimuli, in the early stages of the investigation, the occurrence of this response is usually related to light luminance. If using good autonomous observation viewed the stimulus of light, and at the same time the frequency and intensity of peak were kept constant accuracy; after the stimulus occurred for 12 s or extend to 70 s, the neural system went into a steady state (Regan, 1966). The flash intensity should be elevated higher than the threshold of detection for RGB colors to be sensed by humans (Tolhurst, 1977). It is evidence that RGB color channels exist in human pattern vision of Electrophysiological (Regan, 1974). However, the electrophysiology independent decoding features of RGB color remain unclear, especially on multiple dimensions of time courses and space position.

On the one hand, as RGB color information is constantly changing, the visual system needs the ability to make a selection in time before it is gone. Therefore, the loss of RGB color information can be reduced by using stimuli with a fast speed (Callahan-Flintoft, Holcombe & Wyble, 2020). Steady-state visual evoked potentials (SSVEPs) decoded a visual EEG feature which uses fixed frequency flickering as fast stimuli (Nagel & Spüler, 2019). Moreover, the typing accuracy rate of the SSVEP visual spelling machine which can highly reach 90% (Akram et al., 2013). However, SSVEP also has some major disadvantage, it can not classify non-blinking static targets (Renton, Mattingley & Painter, 2019). If the target contained different colors as the similar blinking frequency, SSVEP cannot supply the efficiently classified color information. In addition, long-term use of flickering images to stimulate subjects can easily cause visual fatigue and eye damage, which will seriously affect the interaction and control effects during the experiment (Bauer, Gerstenbrand & Rumpl, 1979).

On the other hand, the visual system is being able to perceive different RGB colors indicates that there is a specific area of the brain that processes RGB color information. But, this area cannot be located through the cerebral cortex because the EEG features of RGB color information have not been found. Meanwhile, where RGB color information is produced in the primary visual cortex remains unclear.

A kind of theory reflects that color information and other difference evident in sense, is that color information of RGB is not independently processed but in conjunction with other information on the primary visual cortex (Gegenfurtner, 2003). This theory is supported by the joint decoding of image identification technology and brain-computer interface (BCI) (Valeriani, Cinel & Poli, 2017). Graphical decoding can overcome the problem of SSVEP; that is, using graphics can classify and decode non-flickering target. However, graphics are still unable to decode and classify targets of different colors (Min et al., 2016).

Task-relevant joint decoding can help classify targets with the same graphics but in different colors in challenging task. However, the paradigm of task-relevant joint decoding often requires a combination of auditory judgment, psychology thinking, behavior coordination, *etc* (Zhang, Liu & Zhou, 2017). In visual BCI, the most common task-relevant is to ask subjects to find a figure of the target color based on successive prompts and simultaneously tap a keyboard to make a decision (Rohe, Ehlis & Noppeney, 2019). This paradigm appears to be very stable, however, it tends to produce disturbances due to behavior effects and thinking effects (Chholak et al., 2019). In addition, there are too many other reasons for uncertainty, such as insufficient reliability, low classification accuracy, *etc*., these are not very ideal aspects (Kosmyna, Lindgren & Lécuyer, 2018; Kaya et al., 2018; Friedrich, Scherer & Neuper, 2012).

In this work, we investigate a new approach to solve the RGB color neural mechanisms, by analyzing the task-irrelevant random stimuli EEG dataset and at the same time using machine learning combined with visual evoked potential (Petrova, Chepin & Voznenko, 2021). The obtained classification results are combined with an EEG channel to locate the cerebral cortex, and find the position where the RGB color information is processed in the primary visual cortex. The purposes of using this method are as follows: First, unlike the previous task-relevant joint decoding through psychological, decision-making, behaviour *etc*., we ensure that the obtained EEG data only contains visual color stimuli information through task-irrelevant random stimuli. Next, by fixing the luminance parameters in color stimuli to make sure only RGB color information is left in EEG data (Hermannn et al., 2022). At the same time, taking advantage of the high-resolution time-course of fast stimulating EEG enables us to get the temporal features of RGB color information (Lee et al., 2016). Second, the results obtained after testing and training the data through machine learning can quantify the positional accuracy of spatial localization.

The experiments were designed to enable answer two questions: Is it possible to independently decode purely visual color EEG features by stripping task-relevant interference through task-irrelevant random stimulus models? Where are these features specifically distributed in the cerebral cortex? We solved these problems through analyzing EEG data collected from 14 subjects. We used fast active task-irrelevant random stimuli. Using visual evoked potential (VEP) combined with fast active task-irrelevant random stimuli is one method among many for exploring visual EEG signal features (Zhang, Kong & Yang, 2010). The challenge in exploring independent features of RGB color is that features components are complex, especially for co-stimuli performing task-relevant. Analyses of fast active task-irrelevant random stimuli solve this problem because they uncover the pure features of RGB color information after all other interfering information was excluded, and found EEG signal features in both temporal and position dimensions, providing insight into the visual color information processed by the brain (Sato & Inoue, 2016).

The stimuli color space was defined by the RGB color system models (Hebart & Baker, 2018; MacLeod & Boynton, 1979). The RGB parameter adjustment of absolute pure color in red, green and blue can be found in the “Methods” section.

In the above, we have mentioned that in the early research, luminance has a certain influence on the perception of color information by the visual system (Derrington, Krauskopf & Lennie, 1984). Given this factor, it was important for the experiment to have a control variable of luminance, which we ensured by fixing the luminance thresholds for all stimuli to ensure that the effect is minimized for reliable data.

Up to today, there is no experiment to localize cortical regions by using the classification accuracy obtained by machine learning, and this is the first attempt. The results show that when visual stimuli are actively stimulated using the adjusted red, green, blue colors as task-irrelevant random stimuli, using the produced event markers and labels controls the supervised learning classification accuracy. The channel of the visual cortex is as high as 93.7%, and the power reaches the peak decoding within 300 ms after the occurrence of ERP, which has a strong cross-temporal decoding ability. This stimulating approach makes the interactive system interface more extensible, direct, and acceptable to daily needs than earlier systems (Sato & Inoue, 2016). In addition, we report the functional regions of RGB color information processing found by channel position. These regions are EEG data obtained in the same cohort of subjects. The enlightenment of the RGB color information electrophysiological signals space distribution features supplies a little new contribution to providing applications of neuroscience and a non-invasive method of therapeutic.

## Materials and Methods

### Subjects and task-irrelevant

**Figure 1:**
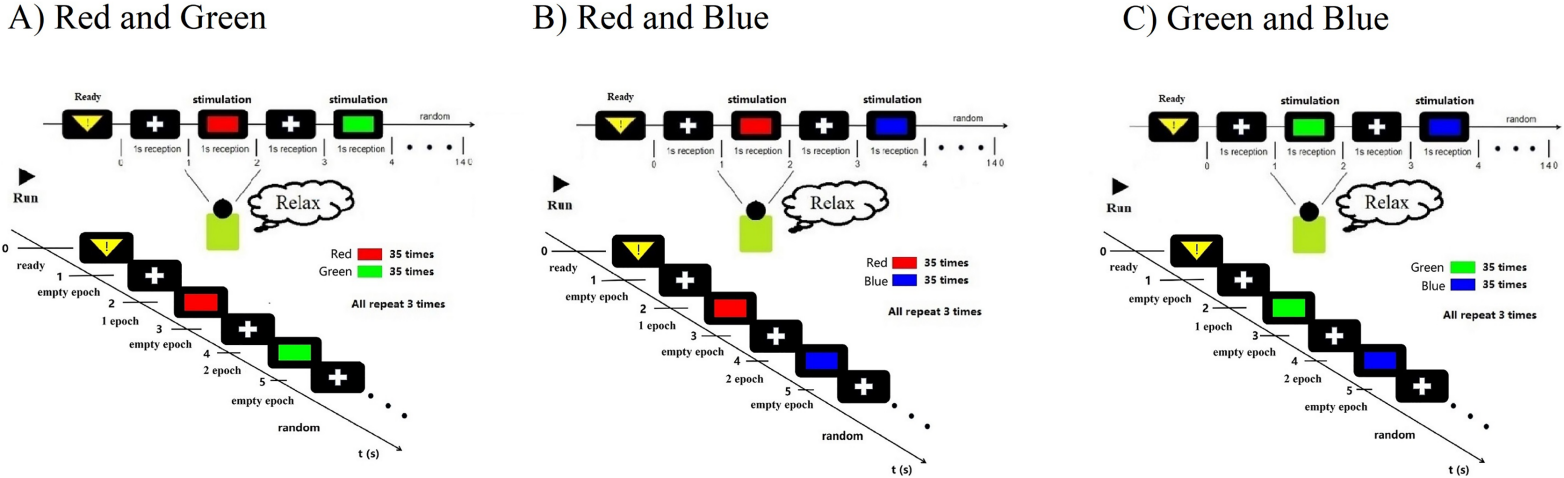
Experimental paradigm. (A) RG team experimental paradigm. (B) RB team experimental paradigm. (C) GB team experimental paradigm. Participants were accepted with EEG data collection while looking at the random stimuli color for 1 s. The colour is one of three adjusted RGB colors. Stimuli were randomly interleaved, with 210 presentations of each stimulus. Empty epochs during eye blinks were removed, and the remaining epochs were randomly generated 70 times per trail. The figure depicts the experimental set-up and illustrates the stimulus interaction flow.

All subjects (*N* = 14, seven males, seven females, mean age 23.2 ± 2.34 years) had normal vision and agreed with the experimental consent. Each subject accepted financial support with $30/h. They all passed colorblindness screening; none of them are visually impaired. None of them had previously been experimented with on the P300. During the experiment, subjects will accept task-irrelevant random fast active visual color stimuli. Task-irrelevant stimuli do not require subjects to perform any additional tasks and do not require any behaviours and decisions that would have delayed effects. Subjects were only required to keep relaxed during the whole experiment when receiving stimulation (Fig. 1). However, to better control the subjects’ self-awareness when recording course, the stimulation paradigm also adopts a rapid, random and active method to assist. Fast stimuli can well evoke great discharge power of visual rod nerve cells within a very short time when the stimulation occurs. Typically, this process appears within a few milliseconds (Barbosaa, Pires & Nunes, 2016). While external visual stimuli can be reflected by P300 within a latency of 300 ms, the color visual interaction latency period remains unclear (Petrova, Chepin & Voznenko, 2021). In this work, epoch refers to each human-computer interaction of a single subject in one team, one trial of experiments. Each epoch contains the time taken for a complete visual color interaction and the corresponding EEG information. ERP indicates the occurrence of evoking stimulus events. Each epoch contains an active stimulus event (ERP) evoked by a random color. All electrophysiological signals converted by the optic nerve emanate through the cerebral cortex and form electroencephalogram signals (EEG). We have recorded the generation and changes of these EEG signals through experiments. Therefore, in experiment each epoch time windows of interaction were set to 1 s. At the same time, the use of random stimuli and fast stimuli can reduce distractions from other aspects during visual interaction. Because subjects can not respond for a short period, it can ensure that the EEG data contains only information on visual electrophysiological stimulation. In addition to random and fast stimulation, active stimuli use the theory based on ERP to record related random events which can be used in supervised learning to decode and classify RGB colors. In each interaction, there was a randomly generated only one event and one label. We defined this interaction for one epoch (Fig. 1). To decode the RGB color through binary classification, the color of red, green, blue are compared in pairs and cross-validated. We define groups of two colors as RG, RB, and GB. In each team, there were three trials. On each trial, a color have an average of 35 random crossover interactions. Two of the colors interacted a total of 70 times in each random trial. One interaction lasted 1 s. There were 70 epochs generated in one trial. A “yellow triangle” image on screen means the stimuli will start. A “white cross” image on screen means rest (Fig. 1). During the time of rest, the subjects can move freely, and the EEG data were not recorded in this period. Finally, all time of one team was 210 s. All three teams of one subject interacted for 630 s.

All experiments involving human subjects complied with the requirements stipulated by the Helsinki Treaty. All experiments were approved by the Ethical Committee of the Graduate School, Fudan University. Subjects supplied informed consent. The subjects informed consent we received during the experiment.

### Visual stimuli

**Figure 2:**
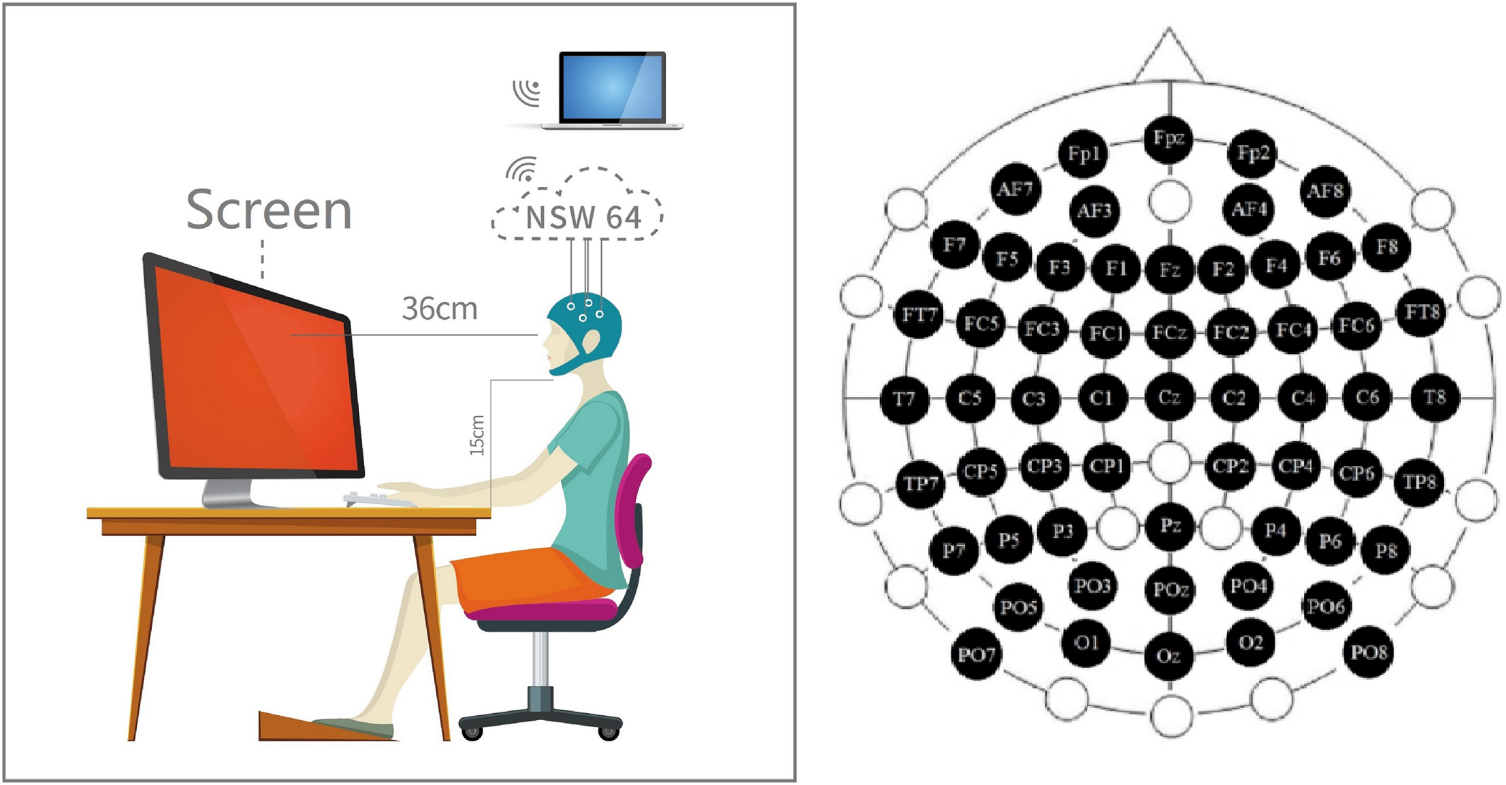
Experiment preparation. Participants performed EEG data acquisition in a dark room. Participants maintained a fixed distance and height from the stimuli source. The channel location distribution of the EEG cap is distributed according to the 59-channel standard. A professional adjusted the cap location before starting the experiment to ensure that the channel is consistent with the anatomical position.

The stimulus was a pure color displayed by the screen after adjusting the uniform brightness (3/5) in an opaque darkroom (Fig. 2 middle four photos just a demo. It is impossible to shoot in the darkroom during the experiment process). The three colors were defined in the Ostwald color space system: this color space is defined according to the 360°, 24-color circle. The three colors were defined by the RGB color space. We fixed the saturability at 100% and fixed the luminance at 50% (Table S1). We designed the color such that (i) the variable in stimulus information vary only around RGB values, and (ii) the effects of interchromatic saturation and lightness were excluded between separate hues. We can approximate that the results of decoding are not to be influenced by other aspects.

### Data acquisition and preprocessing

Subjects EEG data were collected by Start Neural Sen W system. We used Trigger Box designed the experimental paradigm. The raw EEG data sampling at the rate of 1,000 Hz.

After collection, the raw EEG data were preprocessed to remove the effect of head movements, and then use notch filtering to continue to remove the effect of power-frequency noise. Power frequency noise is usually above 50 Hz, we processed the raw EEG data using notch filtering. The EEG signal effective frequency band was usually concentrated in the low-frequency area less than 30 Hz, so high-frequency information greater than 30 Hz was of no value to the experiment, we use a basic finite impulse response (FIR) to filter it out. Next, the data should be downsampled. We resampled the raw data frequency reduced from 1,000 Hz to 200 Hz. The resampling aim was to minimize the uncorrelated association between a few sampling points. After that, we continue processed the EEG data to remove artifacts caused by eye movement to ensure signal purity. Following this, each trial EEG data was extracted and saved by NSW 64 system (−200–1,000 ms around stimulus onset) at the same time removing the baseline mean (Table S1).

### Control the environment to reduce the effect of the experiment

Before starting the prime experiments, we arranged the external environment of the experiment to reduce the impact on decoding. The whole experiment process was in a room without any light to prevent any impact of external light and color. During the whole experiment, the subjects were required to keep relax. At the same time, the subjects were asked to hold their jaws on a shelf (15 cm) and keep a stable sight distance (36 cm) to the screen during the whole experiment (Fig. 2). Then, they can put on the EEG hat (Fig. 2 left inset).

All channel positions on the EEG hat must be checked correctly. In this work, we used 59 channels international standard Fpz-O2 system (the channel ECG, Veol, Veou, Heor, Heol are the signals of electromyography (EMG) which were no use in our experiment) (Fig. 2 right inset). First, we must ensured the CZ channel at the sagittal line’s central. Next, adjusting the CZ channel equal spaces to left/right ears, forehead nasal tip, and inion. The position of the CZ channel must be accurate, and keep the error at a minimum for 0.2 cm.

When EEG data was recorded, a high-level impedance would generate more noise that affects the classification decoding accuracy. We kept the impedance of each channel lower than 3 kΩ (Table S2). Using a medical coupling agent can well reduce the impedance. Before EEG data was recorded, we applied it into each channel. Meanwhile, during the whole experiment, we overseed the impedance of the electrode channel. In addition, the synchronizer was used to reduce the time error of simulation and recording when interacting.

### EEG signal data analysis and processing

The analysis and processing of EEG signal data was carried out using Matlab software. There were 210 empty epochs in the resting time when the screen showed the “white cross”; these empty epochs didn’t have any color information, and they were rejected (over three trials). The experiment was performed continuously in 1-s steps, and we added an empty epoch between two epochs containing EEG color signals. Continuous fast stimulation will make the eyes unable to adapt and adjust in time, and at the same time, disturbance signals will be generated due to eye movement and blinking. The empty epoch sandwiched between two consecutive stimulation epochs with EEG information allows the eyes to rest properly and prepare for the next stimulus interaction. The empty epoch also allows the eyes to adapt and perform physiological actions such as eye movement and blinking during this second. Therefore, the epochs containing EEG information are both independent and intermittently continuous with each other. The empty epoch can directly remove some unavoidable disturbance signals generated by blinking and eye movement from the source during the signal acquisition process, avoiding the trouble of separating from the EEG signal in the subsequent data processing. Because in many previous studies, the disturbance caused by eye movement and blinking will affect the screening of EEG signals, and it is easy to delete the required EEG signals by mistake in the later signal processing process. Other epochs which contained the information of RGB color in three trials were a totally of 210 epochs. To make sure that each RGB color was analysed for the same number in trials, these epochs contained the random colors of two in one team for 105 (team: RG, RB, and GB) (Table S2).

**Figure 3:**
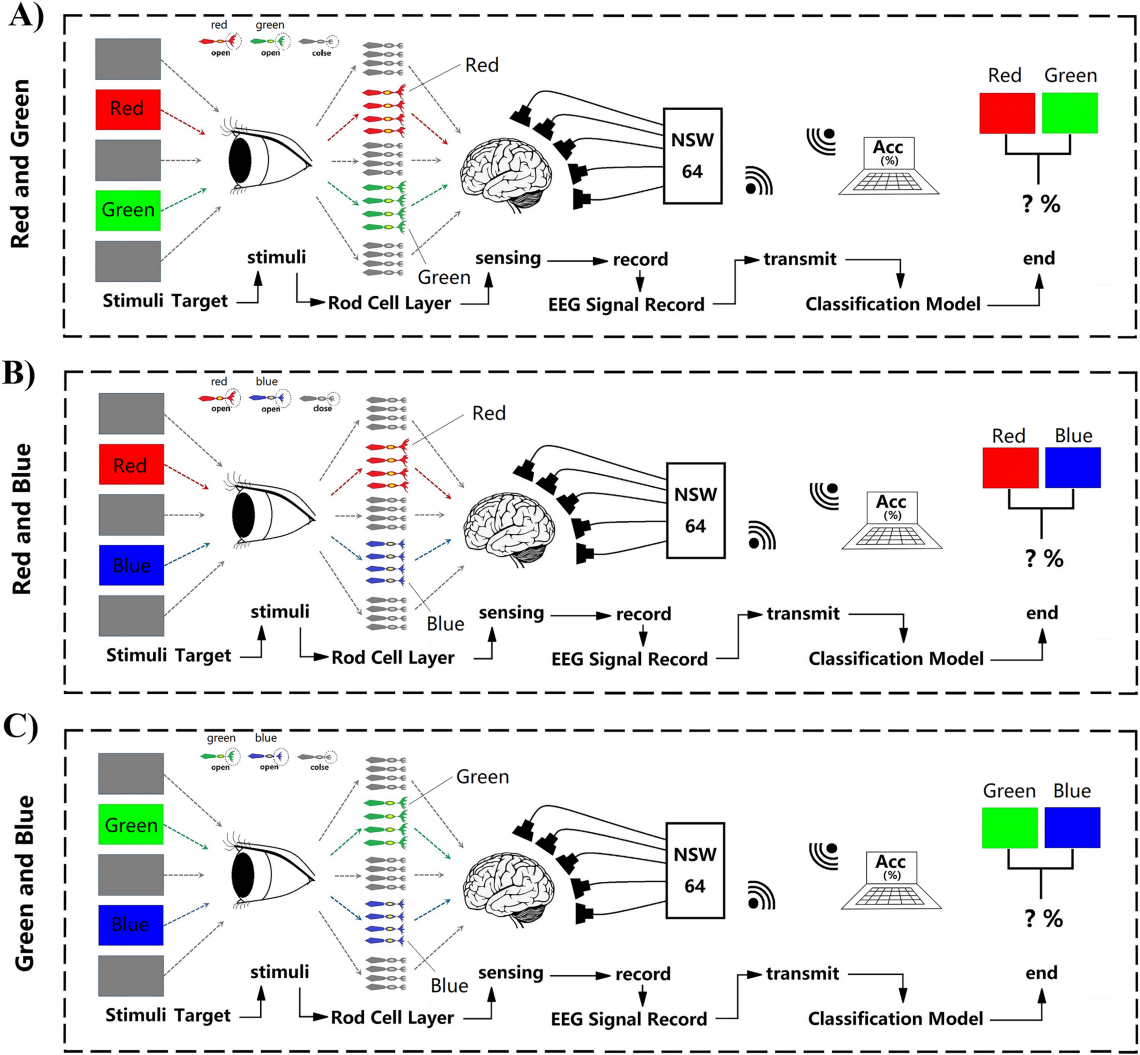
(A–C) Random fast stimuli and EEG data processing.

Two parts consisted of the whole decoding process. First, subjects saw the target, leading the active stimuli signal reach to the rod cell layer, after that transmission to the brain (Fig. 3). When the rod cell layer stimuli information was sensed by the brain, within a short time the nerve endings would discharge great power (Fig. 3). The Start NeuSen W system started recording EEG data. The system produced by Neuracle Technology (Changzhou) Co., Ltd. Next, the NSW 64 system saved EEG signal data and transferred it to the decoding model classifier (Fig. 3).

**Figure 4:**
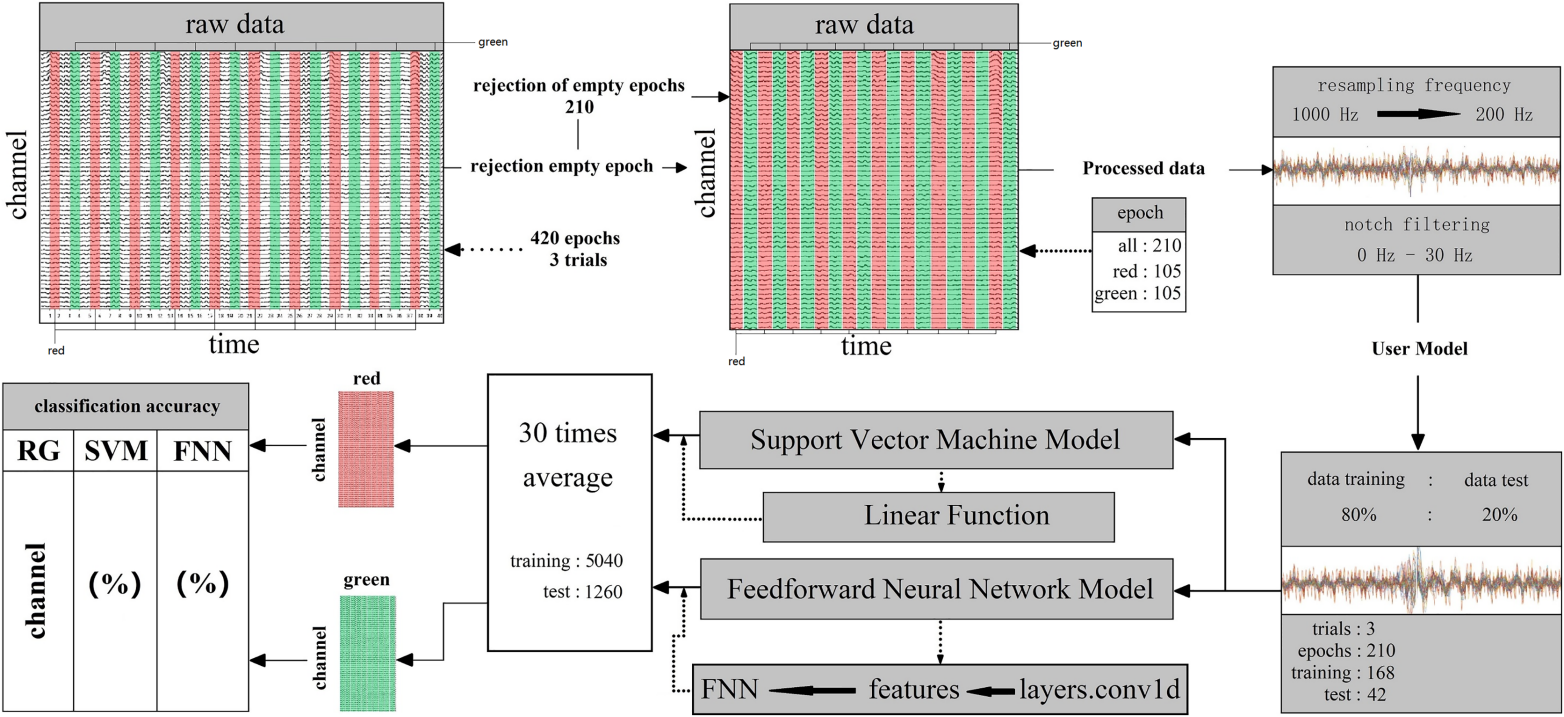
The decoding process of machine learning. The classifier is mainly composed of support vector machines and neural networks. The raw data of one team contains three trails all 420 epochs. Cut the raw data, and reject 210 empty epochs. The remaining 210 epochs contain RGB color information, including 105 epochs for each color. The next step is to preprocess the cut raw data. First, resampling the frequency from 1,000 Hz to 200 Hz. Then, removed the power frequency noise above 30 Hz by notch filtering. Set the ratio of preprocessed data training and test is 80%: 20% and the epochs for training are 168 for testing are 42 in three trails. Put the divided dataset into the main function of the support vector machine and neural network (feedforward neural network) after cross-validations and take the 30 times average as the final result (training: 5,040, test: 1,260).

Machine learning was used binary classification for decoding. Supervised learning based support vector machine (SVM) and feed-forward neural network (FNN) techniques were used to train classifiers on all 14 subjects 59 channels EEG signal data. Linear function and layers.conv1d function was used in the data analysis (SVM) system contains many main functions for kernel function, Gaussian function and linear function. After many comparison tests, in this work, it was more acceptable to use the linear function on binary calssificaiton for processing EEG signal data. In FNN, the data was trained and classified by the neural network; next, using layers.conv1d function to extract the features. At last, for classification, putting the features into the FNN which fully connected (Fig. 4). The training datasets ratio and testing datasets ratio were adjusted for 8:2 (training datasets was 80% for whole datasets, testing datasets was 20% for whole datasets) (Fig. 4). For the EEG signal dataset we collected is a balanced data set and a small sample data set, it is suitable to use HoldOut cross-validation. In this technique, the whole dataset is cut into two sets with training sets and validation sets randomly. Based on experience, usually, 80% of the whole dataset is chosen for training and the resting 20% for validation. The advantage of this cross-validation is that it can be performed quickly: For we must cut the dataset once into two sets one for training and the other for validation, and there will be only one for the model built on the training set, it can be performed quickly. Since our dataset is a balanced dataset, and each group only has a two class (“red” and “green”, “red” and “blue”, “green” and “blue”) in two situations. Taking the two cases of “red” and “green” classes as an example, suppose 50% of the EEG signal data is class of “red” and the rest 50% of the data is calss of “green”. A train-test split was performed with a training set of 80% and a test set of 20% for the whole dataset. It cannot happen that all 50% of the data in the class of “red” is in the training set and all the data in the class of “green” is in the test set. Because the maximum limit of the test set is only 20%. However, in order to avoid some extreme situations, for example, 50% of “red” in the training set, or 50% of “green” in the training set, we need to iterate each result and find an average value. A single classification result is not enough to represent all the results. So our model generalizes well to our test data; since it has not seen the “red” or “green” class of data before; moreover, in the case of small datasets, all samples are used to test the model, so our model will not miss any important features since it was trained on all the data. In addition, iterations number was adjusted for 30 times. When the iterations number over 30 times, the system was overfitted. Therefore, the system fit iterations was 30 times. Time series cross-validation were used in this work. Data is collected at different time points. It were collected in adjacent time periods. We divide the EEG signal data into two sets of training and validation by time. The training were started with a small set of data. The later date points were predicted based on this set and then check for accuracy. Then the training EEG dataset of next contains the predicted samples as one part and forecasts on consecutive samples. In FNN, we use a single-layer network, and function layers.conv1d. Since our dataset is irreversible as the time series moves forward, FNN is very suitable. Because in FNN information also always moves in one direction; it never goes backwards. The kernel we use in SVM is mainly a linear function. It is a polynomial kernel function special case, simplicity is its great advantage, but disadvantage is that there is no solution for linearly inseparable data sets. The linear separable case is its main use. We find that from the feature space dimension to the input space dimension all in the same, and its parameters are scarce and speedy. It is ideal for classification effect to linear separable data, so a linear kernel usually first try to use. If the classification effect of the function is not ideal, tyr to use other kernel functions. To specify the object, the property-value pairs are used in the constructor. After the object is created, to access the values of the object property the dot notation can be used (Table S3). Since our dataset is not high-dimensional, it is not possible to use a kernel such as the Gaussian kernel function for testing. Because the Gaussian function can expand the input feature vector into an infinite-dimensional space. The value calculated by the Gaussian kernel function is always between 0 and 1. Samples can be mapped to higher dimensional space, which will cause the original characteristics of the data to be lost. In addition, due to the small amount of sample data, other kernel functions are not applicable. Finally, classification decoding used the 30 times iterations average value. EEG datasets which contain 210 epochs were cut into 168 epochs and 42 epochs for training and testing (Fig. 4). Whole datasets contained 5,040 and 1,260 epochs in training and testing after 30 times iterations (Fig. 4). By cross-validation got the classification decoding final result (Fig. 4). An epoch is defined as: the smallest and most basic interaction unit, including a one-time random single-color fast stimulus interaction time and corresponding EEG information. Acquired individual subject EEG data are usually stored in .bdf format. The data includes raw data and label data. The label data records the start time of the relevant event (color fast stimulus event) generated by ERP. In this work, we have incorporated related methods for performing data reading while building the model. Data can be read in MATLAB *via* importNeuracle, or in Python *via* MNE.

In FNN, in order to improve the processing efficiency of the model, *N* samples are usually grouped into one team and calculated in batches. Supposing that the input of the first layer of the network is ${A^{\left( {l - 1} \right)}}/in/mathbb{R^{N/times{M_l}}}$, where each row is a sample, the calculation formula of the first layer in the FNN is:

(1) $${Z^{\left( l \right)}} = {A^{\left( {l - 1} \right)}}{W^{\left( l \right)}} + {b^{\left( l \right)}}/in/mathbb{R^{N/times{M_l}}}$$

(2) $${A^{\left( l \right)}} = {f_l}\left( {{Z^{\left( l \right)}}} \right)/in/mathbb{R^{N/times{M_l}}}$$

Among them, ${{\rm Z}^{\left( {\rm l} \right)}}$ is the neurons net activity value in layer *l* of *N* samples, ${{\rm A}^{\left( {\rm l} \right)}}$ is the neurons activity value in layer *l* of *N* samples, ${{\rm W}^{\left( {\rm l} \right)}}/{\rm in}/{\rm mathbb}{{\rm R}^{{{\rm M}_{{\rm l} - 1}}/{\rm times}{{\rm M}_{\rm l}}}}$ is the layer *l* weight matrix, and ${{\rm b}^{\left( {\rm l} \right)}}/{\rm in}/{\rm mathbb}{{\rm R}^{1/{\rm times}{{\rm M}_{\rm l}}}}$ is the layer *l* bias. Here, a tensor of shape (number of samples × feature dimension) is used to represent a set of samples. The matrix *X* of samples is composed of *N* row vectors of *x*. In order to make the subsequent model building more convenient, we enhatsulate the computation of the neural layer into operators, and these operators all inherit the op base class.

All classification models can be run under MATLAB 2019A and Python environment. Codes of SVM and FNN can be found in Science Data Bank. All results in the manuscript can be reproduced.

## Result

**Figure 5:**
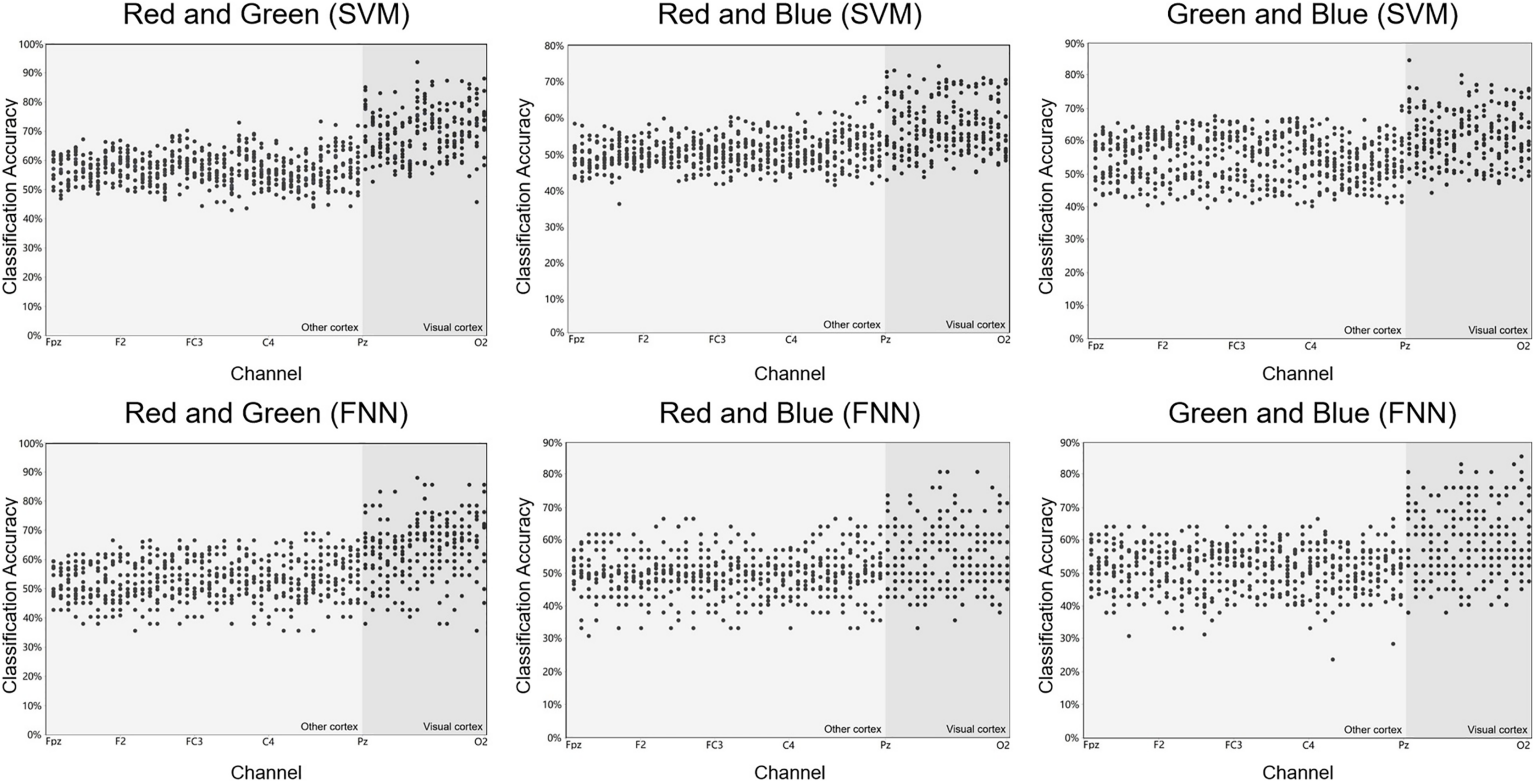
Raw results of classification decoding. ABC shows the raw classification decoding results obtained under the two classifiers for the RG team, RB team, and GB team with 14 participants. The figures show the whole cerebral cortex and all 59 channel classification results of each participant. Preliminary analysis showed that the channels with higher classification accuracy were generally concentrated in the primary visual cortex (dark grey background). The highest classification accuracy channel obtained under both classifiers in each group was also present in the primary visual cortex (dark grey background). The channel classification accuracy rate in the visual cortex is generally around 70%, a small part is around 80%, and few can reach around 90%.

We used SVM and FNN as a classifier. We performed within-subject decoding (trained and tested on EEG data for each subject, training set = 80%; test set = 20%), independently for each epoch (applied independent classifiers at each epoch relative to stimulus onset) (Fig. 4). All analyses involved pairwise comparisons and were cross-validated (number of validation = 30 times, take the average of 30 times as final results), yielding plots that show distribution features of the classification accuracy results on the channel (Fig. 5). subjects were told to maintain fixation throughout stimulus presentation and to blink at designated times.

The three colors were random presented. Two of the three colors were randomly cross-combined into one team to ensure that all colors participate in the binary classification task. Each team was into three trials. Each trial consists of 70 epochs. One epoch contains EEG data of one visual interaction by fast active task-irrelevant random stimuli. At the same time, the EEG data also contains information such as latency time features and power peak features after stimulation. Visual interaction time in each epoch is 1 s (1 s inter-stimulus interval). We collected all epochs of each trial of each team (*N* = 630), removed power frequency noise by notch filtering and resampled to preprocessing data of three trials per team. The same stimuli elicited separate data and tested the classifiers. Results disclose the accuracy of classification for RGB color information taken by a particular specific color.

In other analyses, we determined whether classifiers on EEG channels could decode RGB color information generated in the primary visual cortex. The distribution of position provides a result of invariance: the features of classifier decoding accuracy on channel position are invariant to the features of power discharge on channel position.

### Decoding RGB color classification feature

**Figure 6:**
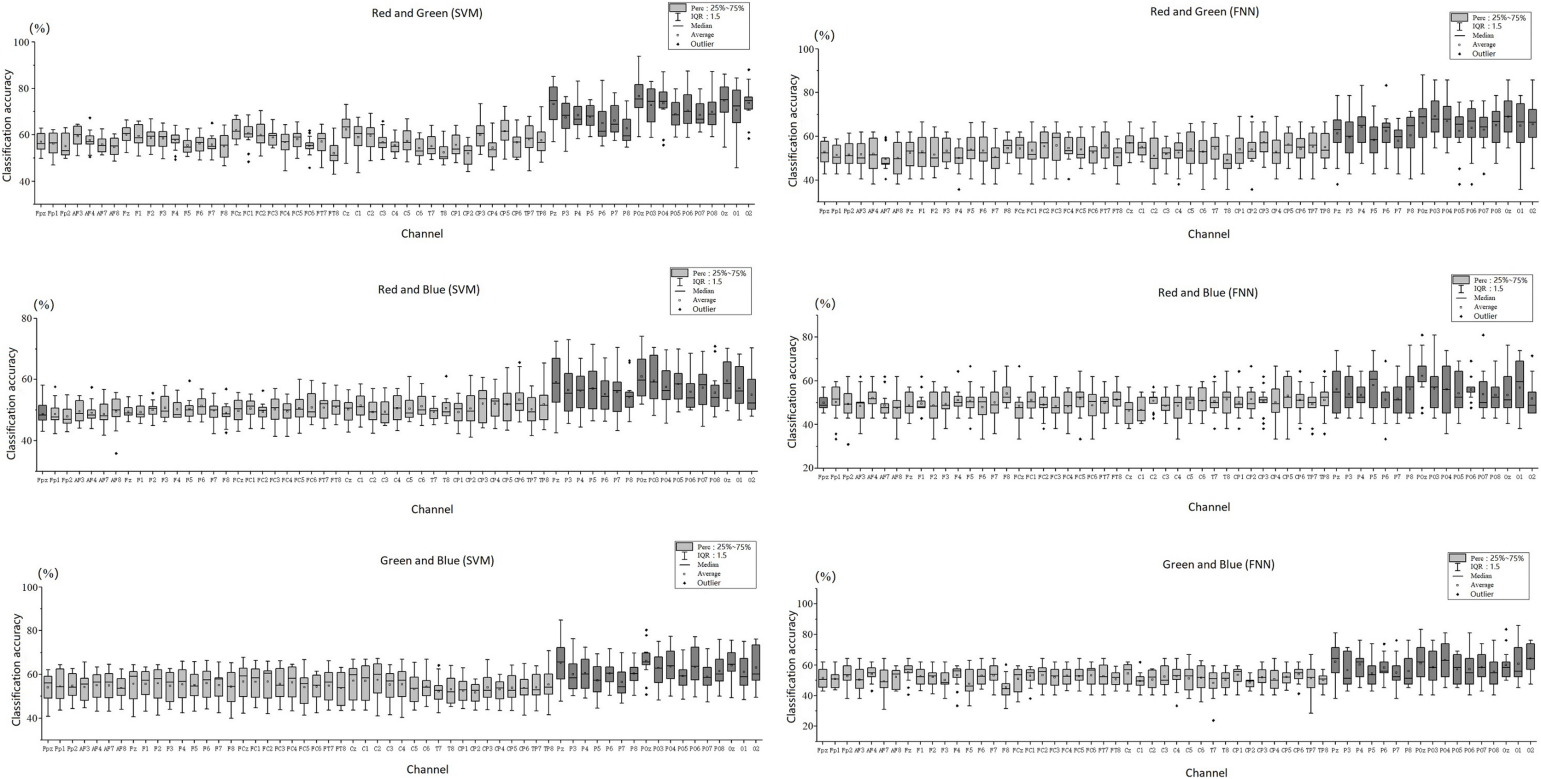
Decoding RGB color EEG data by machine learning. ABC shows classification accuracy across all 59 channels for the three teams (RG, RB, and GB). The classification accuracy results for the 14 participants in each channel are presented as boxplots. Excluding outliers with larger dispersion, the box perc is 25–75%, the range within 1.5I QR, and the average and median (Q2) of the cortex are obtained.

Figure 5 shows the scatter distribution of RG, RB, GB classification accuracy on 59 array channels distributed on the cerebral cortex using machine learning decoding. The consequence display that RGB color EEG can be decoded by SVM and FNN. When the time window of each epoch was cut to 500 ms after the stimulus, the results with higher classification accuracy were mostly distributed in the primary visual cortex (dark grey in Fig. 5). Figure 6 shows the specific performance of the classification results. We performed statistics on the EEG data of 14 subjects using boxplots. Each vertical line, it contains the classification accuracy distribution range of the same channel in the cerebral cortex of 14 subjects. The top of the vertical line represents the highest classification accuracy obtained by this channel (maximum) and the bottom represents the lowest classification accuracy obtained by this channel (minimum). The dot in the centre of the vertical line represents the average classification accuracy of the 14 subjects of this channel. The length of the vertical line represents the magnitude between the highest and the lowest classification accuracy extreme values of this channel. We divided the data into quartiles (perc: 25–70%), that is the box contains 50% data of one channel. The diamond-shaped symbols represent individual outliers with a large dispersion degree. The range of IQR is within 1.5. The horizontal line in the middle of the box represents the median Q2, Q1 is the box upper edge, Q3 is the box lower edge. We found that after removing the more discrete values, the rest of the 50% data in the valid box reflected that both the Q2 and the average value (dark grey area in the figure) were higher in the primary visual cortex than in the other cortex (Fig. 6). In the primary visual cortex of the RG team, box, Q2, and average positions were around a value of 70%; in the other cortex around 60% (Fig. 6A). In the primary visual cortex of the RB and GB team, box, Q2, and average position values were not as high as those in the RG team, but also around 60%; in the other cortex around 50% (Figs. 6B and 6C). It is important to note that the classification accuracy size does not offer an effect on absolute size weigh, but it is still a comparative effect dimension valid method in a certain research (Sato & Inoue, 2016). By comparison, it was found that the values of the primary visual cortex were slightly higher than other cortexes.

**Figure 7:**
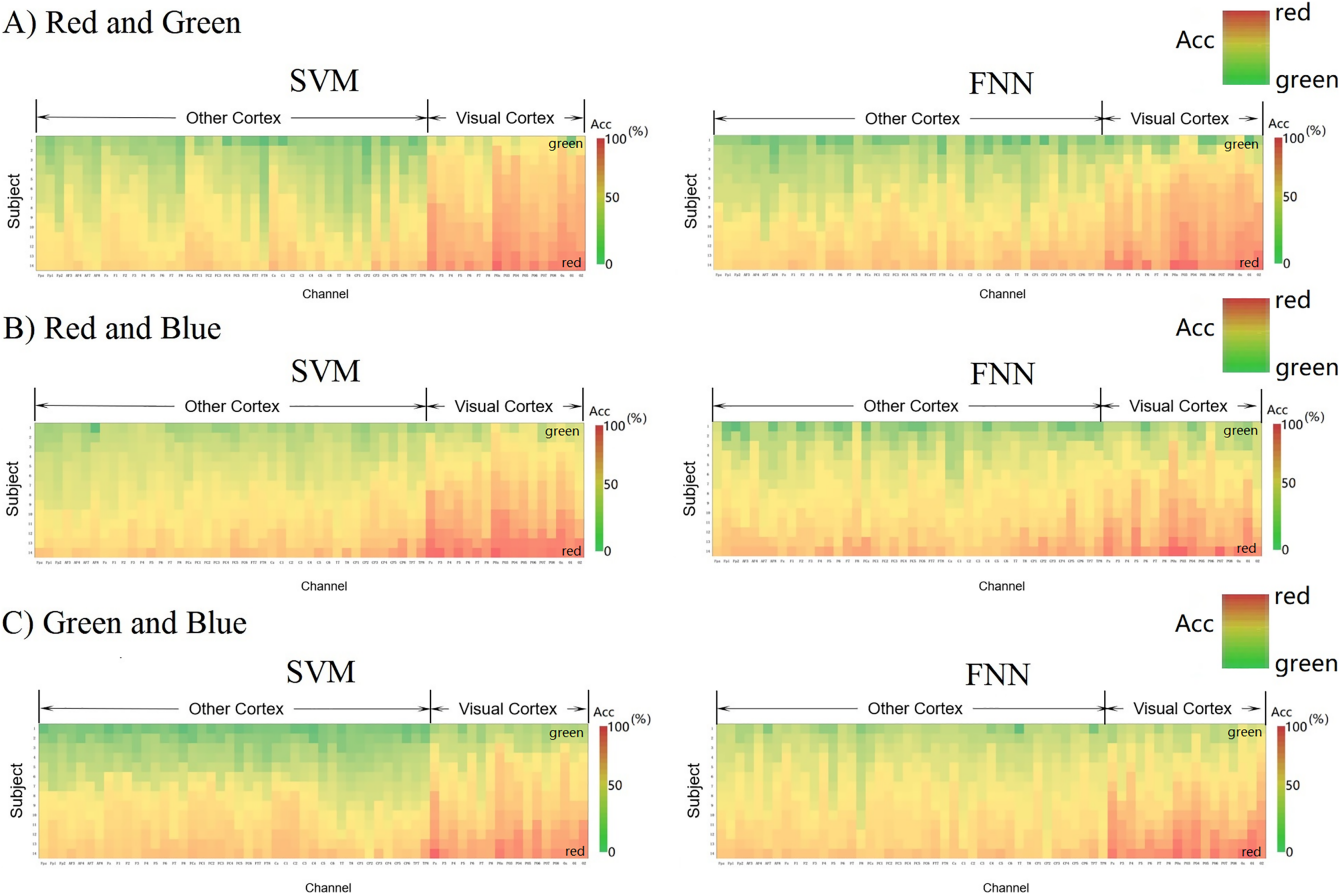
(A–C) Decoding 3D matrix heatmap. EEG data constructs new 3D matrices from 3D vectors. The horizontal dimension of the matrix shows the arrangement order of the channels from the other cortex to the visual cortex. The column vector of the matrix shows that the participants are arranged in a gradient manner according to the classification accuracy of the third parameter. The size of the classification accuracy of the third parameter is represented by the depth of the color.

Let us use another method to intuitively compare the channels classification decoding results of the primary visual cortex (17 channels from Pz-O2) with the other cortex (42 channels from Fpz-TP8). Figure 7 shows each channel decoding classification results through a matrix heat map. The column vector of the matrix represents the 14 subjects in the experiment, the row vector represents the localization channels (59 channels from Fpz-O2) distributed on the cerebral cortex, and the color depth in each square represents the size of classification accuracy value (red > 70%, yellow 50–60%, green < 50%). We formed these three parameters set into three-dimensional data (3 × 3 data) and constructed a three-dimensional space matrix (3 × 3 matrix). In the column vector direction of the matrix, the classification accuracy is sorted according to the size of the gradient, and it is found that most of the regions are yellow (50–60%), which indicates that almost all EEG data are effectively classified by SVM and FNN. The red regions representing high classification accuracy are almost concentrated in the primary visual cortex (>70%). This result shows that RGB color information can be well classified and decoded in the primary visual cortex.

**Figure 8:**
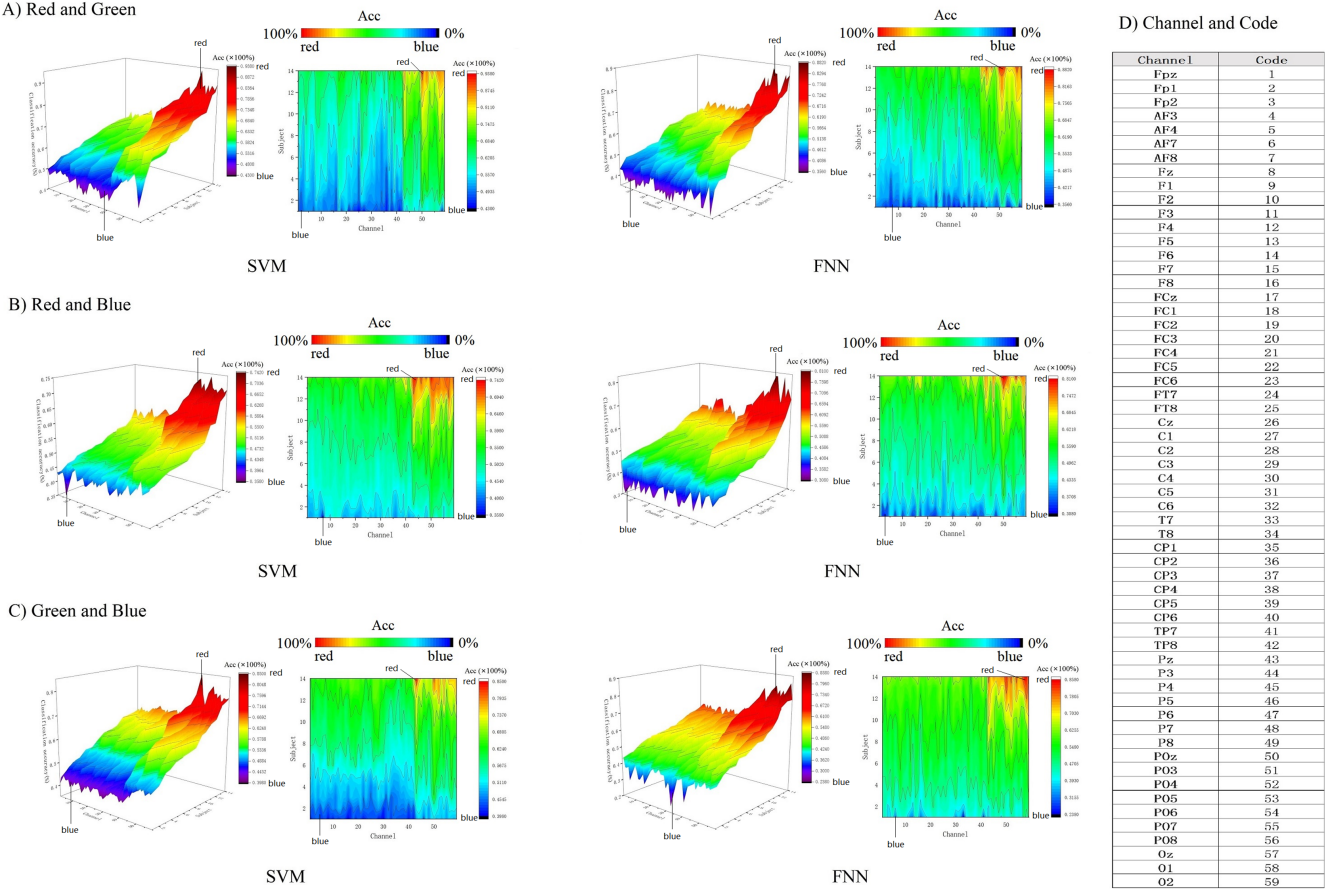
(A–C) Decoding RGB color information by 3D topographic map.

In addition, we use 3D color topographic map that stereoscopically rendered the 3D matrix (Fig. 8). 3D color topographic map more intuitively shows all chanels classification accuracy distribution. The 3D color topographic map on the left side of Fig. 8 shows the channel accuracy of classification gradient change from other cortex to visual cortex on the X-axis (channel axis). Starting from channel No. 43, the classification accuracy has a steep upward climbing trend to the last channel No. 59, and the classification accuracy shows a positive increase rate and reaches the extreme value (see Fig. 8D for the corresponding code of the channel). Channel No. 43 is a border between the primary visual cortex and the other cortex. Therefore, it can be intuitively found that the decoding effect of classification accuracy starting from the primary visual cortex has strong robustness. We further obtained contour maps by Fourier transforming the 3D color topographic map (Fig. 8 right inset). The results are consistent with the matrix heatmap shown in Fig. 7. Therefore, we can summarize the result: channels with higher classification accuracy are concentrated in the primary visual cortex.

**Figure 9:**
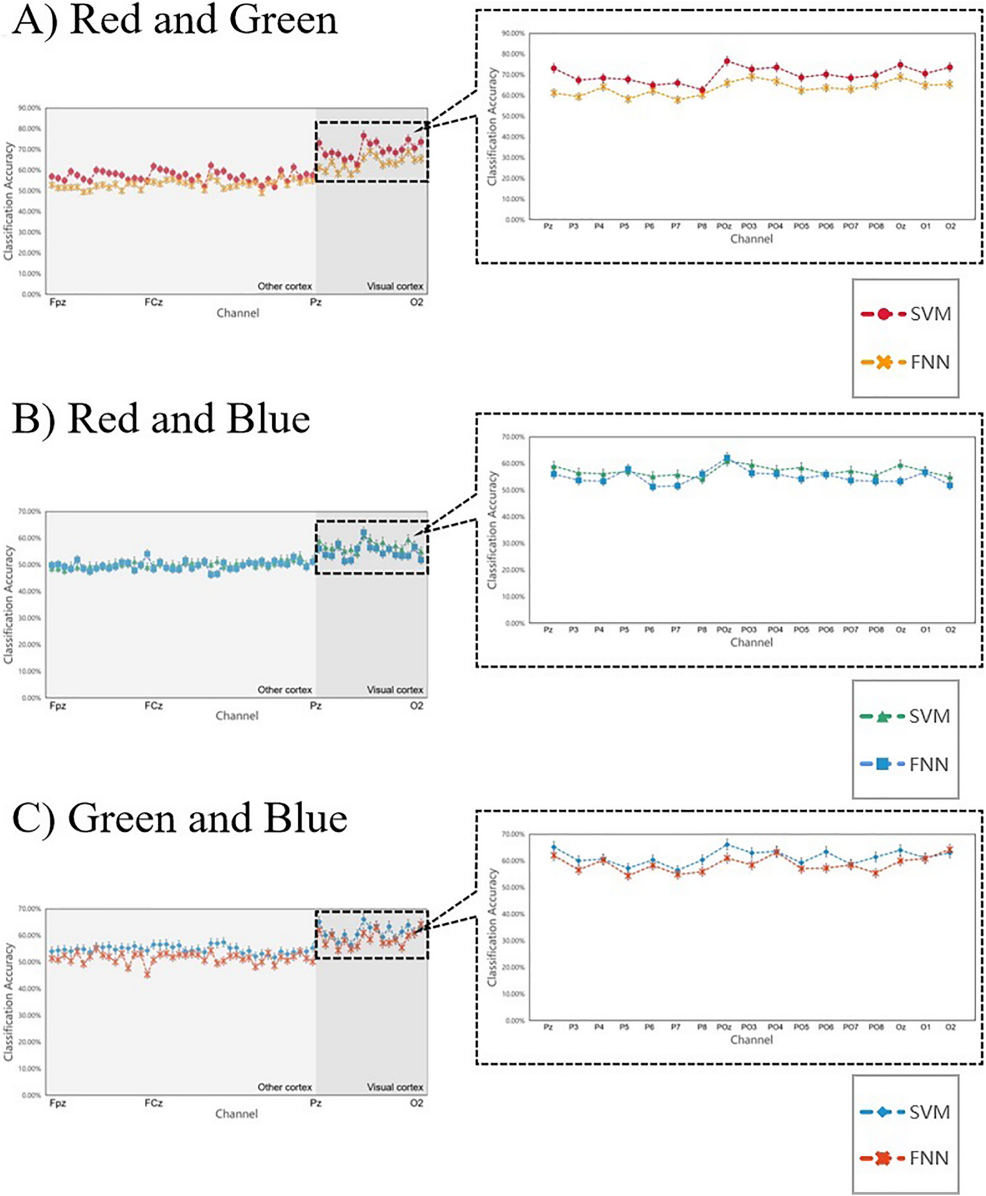
(A–C) Comparing the SVM and FNN decoding results. In each team, the two curves represent the average line of classification accuracy obtained for all 59 channels under SVM and FNN. The fit of the two curves firstly represents the difference in the results, and secondly, whether the decoding results obtained by the classifier are reliable. The right inset shows the classification accuracy of the primary visual cortex areas we are more interested in.

Figure 9 shows the 14 subjects’ average classification results in comparison to SVM and FNN, the results are similar, indicating that the two classifiers have little error and the results are reliable. At the same time, we also analyzed the primal visual cortex with higher classification accuracy, it was found that the results obtained under the two classifiers were the same (in each team the two curves fit) (Fig. 9 right inset). This result suggests that the primary visual cortex is active when the brain processes the RGB color information sensing. Therefore, we can conclude that it is possible to interpret RGB color information through machine learning. These conclusions sustain a hypothesis: the brain can process RGB color information independently in the surrounding task-irrelevant decoding.

**Figure 10:**
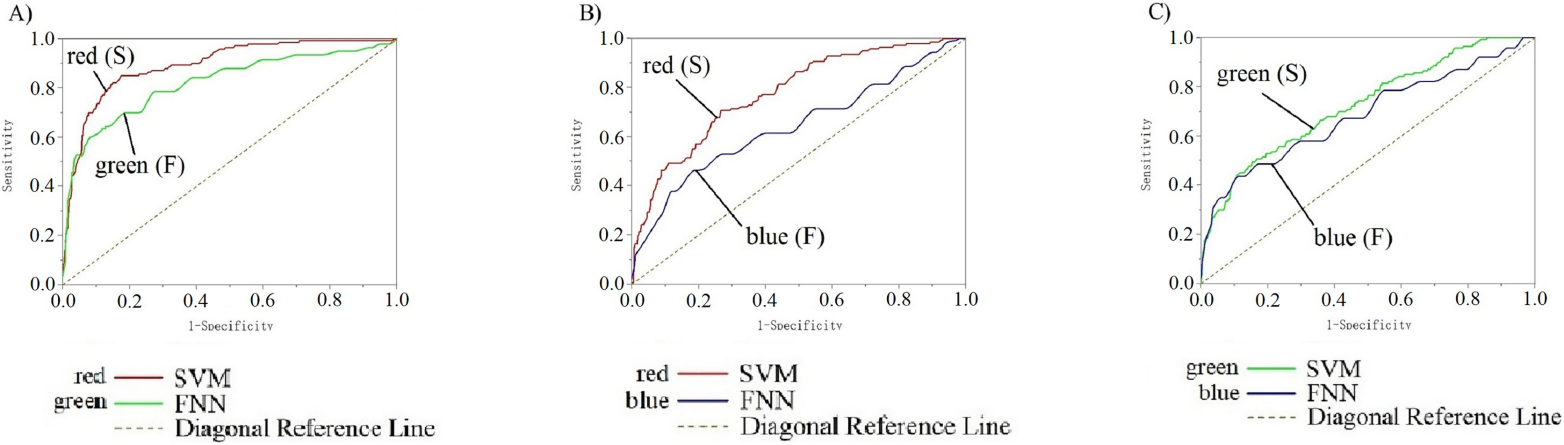
(A–C) ROC curve of 14 participants on SVM and FNN. (A) shows the ROC curve of RG team, red line is SVM and green line is FNN. (B) shows the ROC curve of RB team, red line is SVM and blue line is FNN. (C) shows the ROC curve of GB team, green line is SVM and blue line is FNN. The table below for each team represents the area under the curve. The table to the right for each team represents the state.

Figure 10 shows the parameter analysis on different machine learning models. The ROC curves (receiver operating characteristic curve) show the data training and data testing decoding results with SVM and FNN from EEG collected on 10 channels of the primary visual cortex of 14 subjects. In each team, there were included all 826 data. We divide 826 data to test the two classifiers’ performance and carry out statistical significance testing. Each team input data range is 826. Smaller and larger test measurement values respectively indicate a positive tests and a biased test. Channel POz-O2 is the actual positive state (all: 826; positive: 140; negative: 686). Standard error is 95%, and confidence levels is also 95%. The direction of test is positive v.s. high. Diagonal reference lines are included in results. In the input data, there are no missing values, and the bad data and invalid values are not used in calculations The RG, RB, GB (SVM and FNN) ROC curves analysis results were listed in Table S4. Analysis results indicate that AUC of SVM and FNN in RG: 0.89 > 0.5, 0.82 > 0.5. In RB: 0.78 > 0.5, 0.64 > 0.5. In GB: 0.73 > 0.5, 0.69 > 0.5. The results all over 0.5 have a valid accuracy (Table S4). The two curves sensitivity and 1-specificity are biased towards the diagonal reference line upper left, which means our two models have a bigger examination accuracy. The two curves are fitting means the model results are similar and stable.

### Cross-temporal decoding for visual color ERP

**Figure 11:**
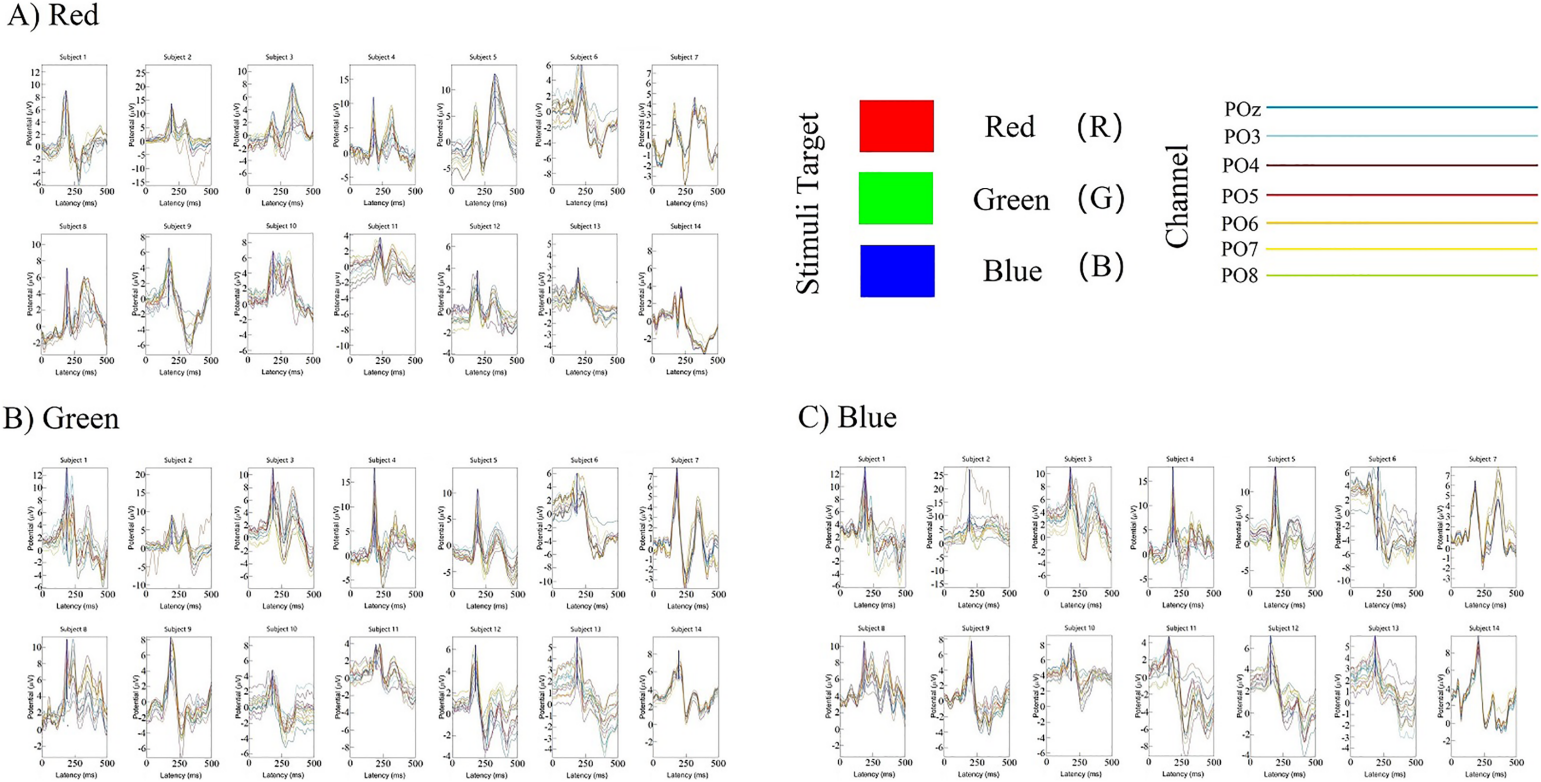
(A–C) Cross-temporal decoding for visual color ERP. ERP subgraph shows the time course of VEP for each participant in an epoch. The horizontal axis shows the time course after the occurrence of the stimulus (mainly showing the latency time distance from the occurrence of the stimulus to the generation of the visual evoked potential). The vertical axis represents the location of the first peak power after stimulation based on the P300 theory. Each curve represents the epoch in three trails for a channel.

**Figure 12:**
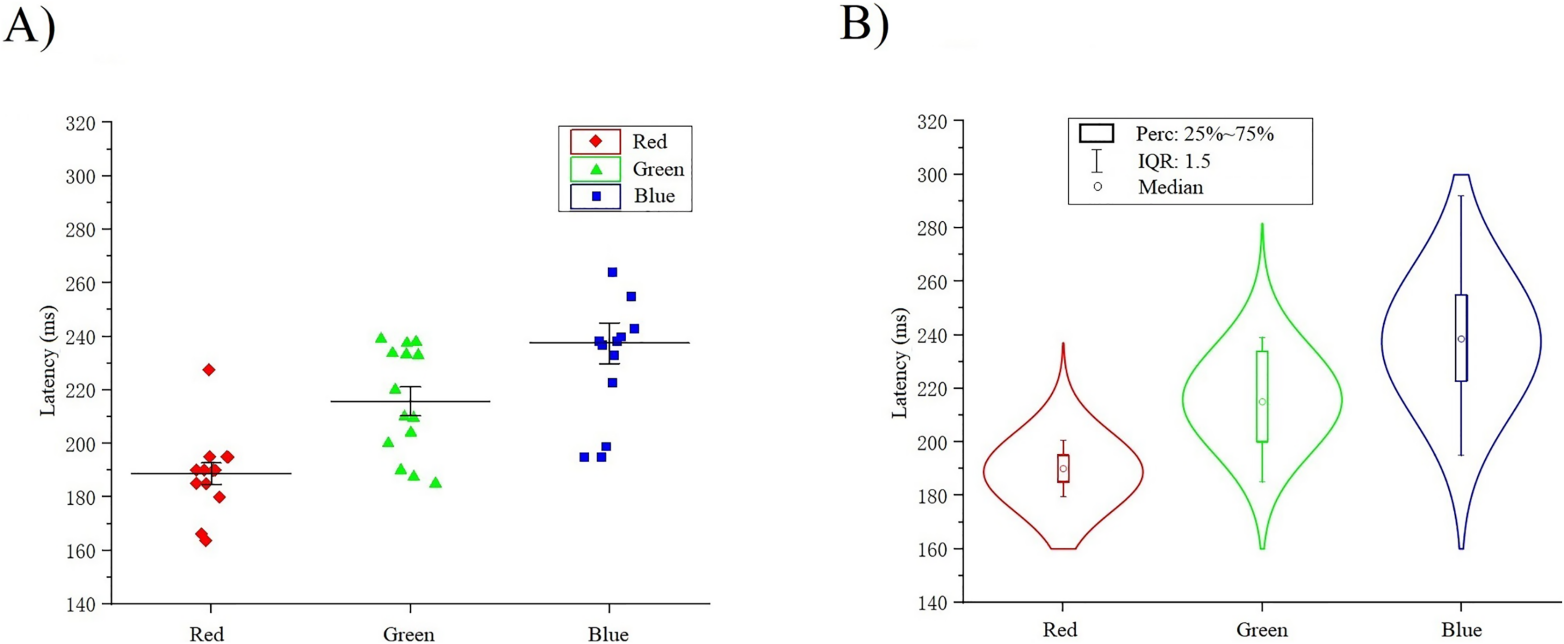
(A–B) Cross-temporal decoding for visual color ERP. A continuous scatter distribution.

The classification decoding results we have discussed so far have been based on evaluating the performance of the classifier using test data obtained at the same period after stimulation as training. The classifier showed significant decoding performance within 300 ms after stimulus onset. The possibility of this phenomenon arises is that the stimulation of RGB color-related events evokes transient activity patterns of visual potentials (Cherepanova et al., 2018). To decode the time course features of transient activity patterns, we performed visual and statistical analyses of the EEG data. Figure 11 shows ERP plots of 14 subjects’ with red, green, blue stimuli in 500 ms on primal visual cortex 17 channels (Pz-O2). Each curve represents a channel. The size of the peak shows the average result of the optic nerve cell firing voltage for all epochs of the same color. We usually think of P300 as an endogenous component of ERP (King & Dehaene, 2014). Therefore, the position of the peak mapped on the time axis (horizontal axis) in Fig. 11 is also an endogenous component of ERP, which is consistent with the P300 theory. The cross-temporal distribution features of all VEPs can be found from the peak-to-time correspondence in Fig. 11: The peak of power appeared within 300 ms latency after stimulation. We performed sample statistics on ERP time points drawn from 14 subjects. All ERP’s 300 ms scatter distribution was arranged in three teams of red, green, and blue (Fig. 12A). Figure 12A shows these three colors in each time segment are concentrated at similar time points. Three teams are distributed continuously. However, some data have a larger degree of dispersion (The reason for the larger error is mainly because the individual differences of some subjects are too large or generated during the experiment). To exclude the influence of these small outliers, we use the method of median statistics. Since the median is obtained by sorting, changes in some data do not affect it. When the individual data in a group of data changes greatly, it still can describe the central tendency of this group of data. The data was cut into quartiles and used boxplots and violin plots to present them. Box perc: 25–75%; IQR is within 1.5. After sorting obtained red: Q3 = 185 ms, Q1 = 195 ms; green: Q3 = 200 ms, Q1 = 233.75 ms; blue: Q3 = 222.75 ms, Q1 = 255 ms. The time segments of RGB color latency are red: 185–195 ms; green: 200–233.75 ms; blue: 222.75–255 ms. Time segments are distributed continuously, and the boxes are also distributed continuously (Fig. 12B). Figure 12B violin plot further shows that the data has a larger kernel density near the median (Q2), indicating that the distribution of data near the median (Q2) is probability larger (the distribution of kernel density is normal). Finally, the cross-temporal decoding results are obtained by performing median (Q2) statistics on the three-time segments of RGB colors: red latency (Q2) = 190 ms; green latency (Q2) = 215 ms; and blue latency (Q2) = 238.5 ms (Fig. 12B the point in the middle of the box).

This result suggests that it is feasible for independent decoding RGB color across time courses based on the theoretical mechanisms of ERP and P300. In addition, the research result indicates that at the time the brain senses the stimuli from RGB color, red was the shortest evoked, then green and blue. It also reveals why human visionual is so sensitive to red. This can also be used to explain why red is often used to inform and remind others of danger (Acqualagna, Treder & Blankertz, 2013).

### Comparing the spatial position of classification decoding and power distribution

To directly compare the space position of classification decoding and power distribution, the 59 channels international standard system were used (Fpz-O2) (Engel, Zhang & Wandell, 1997) (EMG are not used in this work) (Sitaram et al., 2017). Our goal is to use the results of classification accuracy and power distribution obtained from EEG data channels to reverse the calculus of their position in the cerebral cortex. We note that RGB color information exists in the primary visual cortex through previous classification experiments. Meanwhile, the cross-temporal course independent decoding of VEPs shows the features of power distribution. Therefore, we also need to use a simple random sampling analysis of 14 subjects to account for the correlations between classification decoding and power distribution. By comparing the correlation between these results to determine whether these features all come from the same channel position.

**Figure 13:**
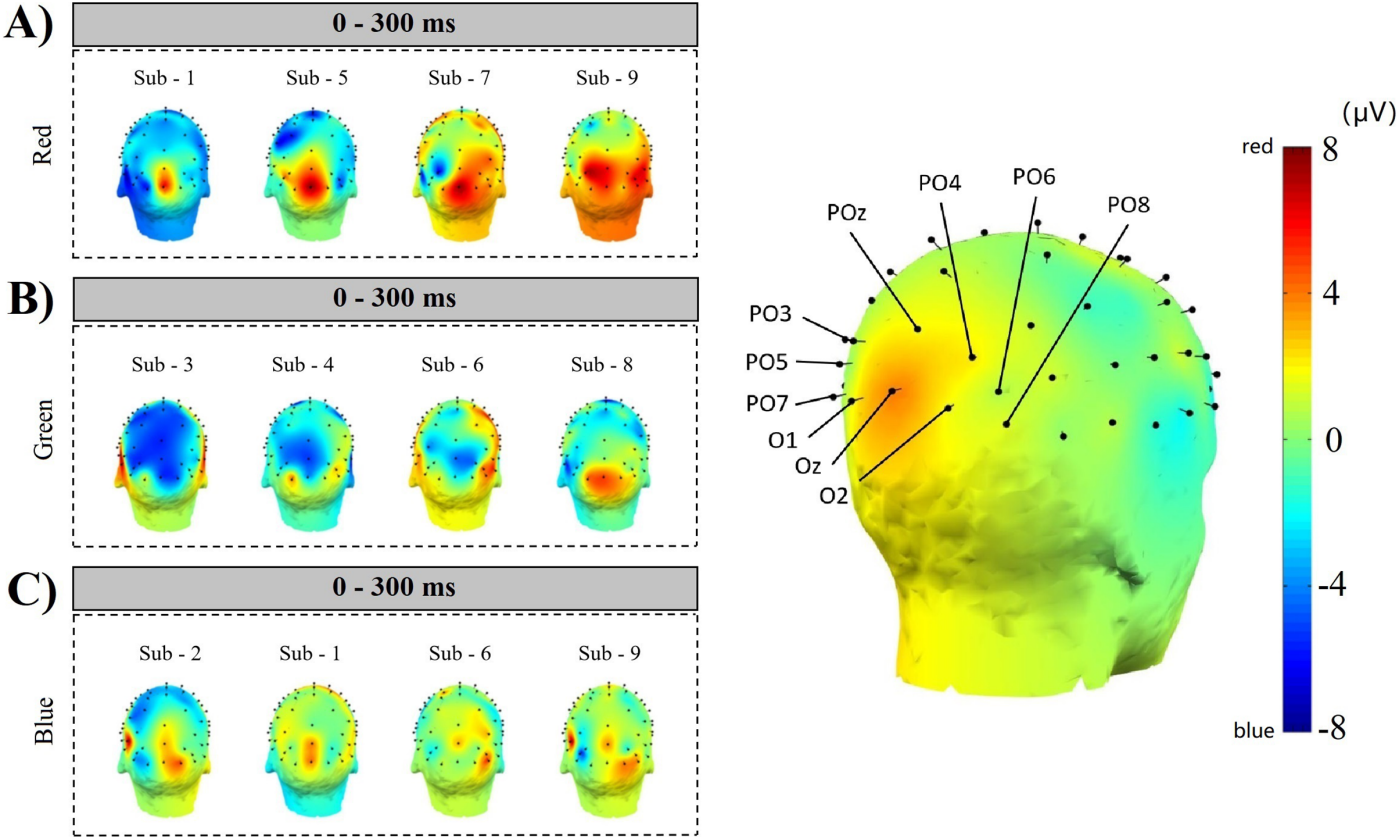
Space location of classification decoding and power distribution. The left inset shows the results of a simple random sampling of 14 participants. The power topography map shows the distribution of the neuronal transient discharge in all 59 channels during the 300 ms latency after stimulation. The results showed that there was a peak of power in the primary visual cortex. The right inset shows the channels that achieved higher classification accuracy over the same time course. Comparing the two illustrations on the left and right, it can be found that when the time course is within 300 ms, the power distribution features of nerve cells are the same as the classification features of machine learning. The three dimensions of the time course, power distribution, and classification accuracy constitute a space feature of decoding RGB color.

**Figure 14:**
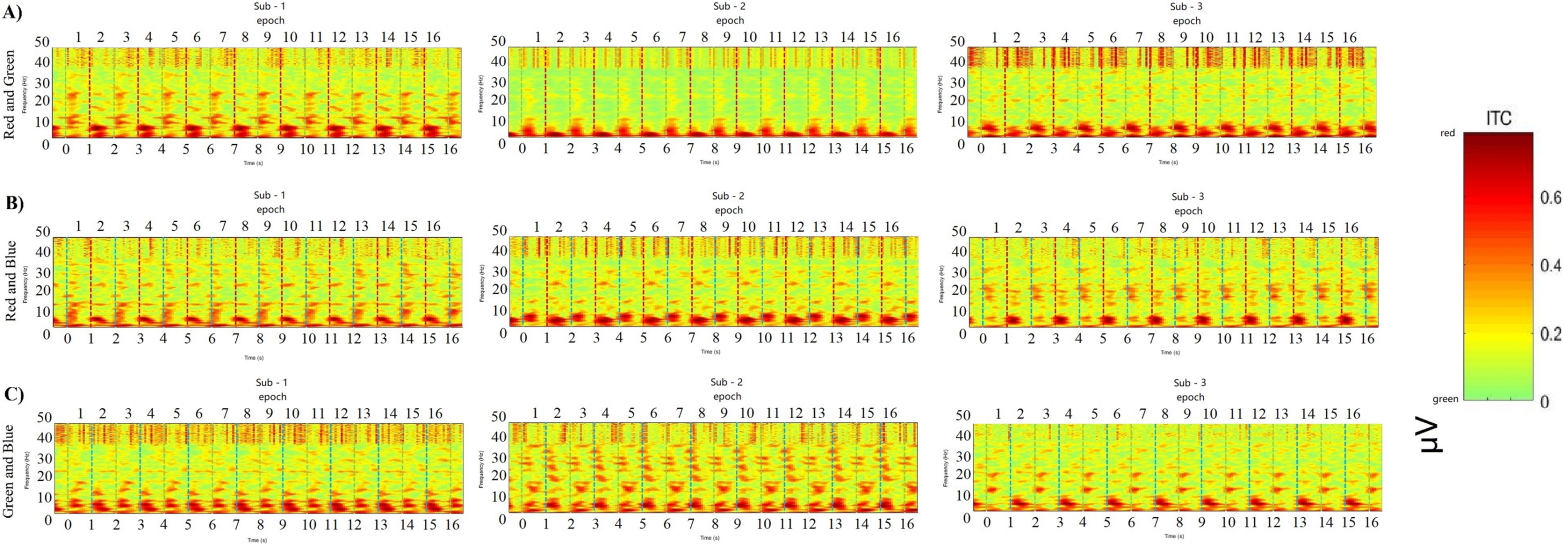
Comparing decoding features for consecutive epochs. ABC shows the results of the POz channel with the best classification accuracy performance from nine participants by a simple random sample. Time-frequency for 16 consecutive epochs shows a regular distribution of power after each interaction appears. The power peaks in the low frequency (30 Hz), and the peaks of adjacent epochs are the same, showing a robust regular distribution.

Figure 13 ABC shows the comparison results of 4 subjects’ simple random sampling analysis (*N* = 14, *n* = 4, 4/14): the 3D topographic heat map (use MNI coordinate file for BEM dipfit model) shows the distribution of power, which is evident in the primary visual cortex; darker areas are found in the primary visual cortex (Fig. 13 right inset, with the channel POz-O2, are prominent), confirming the previous classification and decoding results. We also generated time-frequency plots of 16 consecutive epochs of POz channels with high classification accuracy and power distribution (Fig. 14, random sample of nine subjects).

EEG signals localized to channel POz achieved high results in both machine learning classification and VEP power discharge. The frequency, time and power parameters 3D data spectrograms composed of short-time Fourier transforms (STFTs) exhibit strong correlations. These epochs are arranged at certain intervals according to time series and different color labels. The power discharge is concentrated in the low-frequency region (<10 Hz), and some high power appears within 300 ms of each epoch (0.6–0.8 μV, Fig. 14). These results provide potential correlations between classification accuracy decoding, power distribution, and time course.

Comparing the correlation results of space position, we can get that, first, in the primary visual cortex, RGB color can be independently decoded, and it is easier to decode in the POz channel; Second, the decoding results are interoperable and consistent, which can be cross-validated by the channel space position. Third, this is also the position where the neural representation of RGB color appears.

The distribution features of space position reveal the brain processing RGB color information neural mechanism. We explain the results of position distribution as neural representations evidence in the brain for visually received RGB color stimuli, and we will return to this in the discussion below. The latency to the peak value of decoded RGB color EEG power is often affected by the designed task. Task-relevant sequential stimuli will impact the information’s feed-forward flow (Zhang & Luck, 2009), and after 200–300 ms stimulus the task effects typically emerge onset (Hebart et al., 2018; Grootswagers et al., 2021). Constant with the task effect late reach, EEG feedback from RGB color stimuli was disrupted at times <300 ms. Compared with task-relevant random stimuli, using task-irrelevant random stimuli can obtain purer EEG information. In visual sensing, the extent of the latency incarnates the differences in stimuli perception and classification, and more perceptually different stimuli can be decoded at a later time, and are compared to those performed by regions further down the visual processing hierarchy calculation associated (Proklova, Kaiser & Peelen, 2019; Carlson et al., 2013; Cichy, Pantazis & Oliva, 2014). And the latency of ERP power to reach the peak value caused by the perception of the stimuli was less than 300 ms. Within the same time window (the interaction time window for an epoch is 1 s), the results of stimulus perception and classification are the same, implying that the neural mechanism of RGB color is therefore consistent with the hypothesis that the brain can independently receive and process RGB color information. There are functional areas in the cerebral cortex that can independently perceive and decode RGB color information.

## Discussion

The experiment here produced three results: first, RGB color EEG data can be directly and independently decoded. RGB color EEG data can also be decoded in the task-irrelevant stimuli; Second, the time course features of RGB color decoding reveal that the hypothesis based on the ERP and P300 theoretical mechanisms is valid. The peak value of neurophysiological discharge occurred within 300 ms after the RGB color stimulus. Third, the channels with better classification accuracy and higher power discharge are common channels. The power peak value and high classification accuracy are concentrated in the primary visual cortex (channel POz, Oz). Control experiments show that all results are reflected in the same space channel position. All in all, the results have great important effects on us understanding of RGB color EEG-independent features and how neural circuits encode these features.

It is still unclear what constitutes the visual electrophysiological signature that determines the decoding performance of RGB color classification. However, the results discovered here suggest that (1) The time course is the first feature to be encoded, and (2) the transient power spikes of neuronal cell firing occur in the primary visual cortex. The relative time to decode RGB colors may be relatively small, just hundreds of milliseconds, but it is not only robust, evident within individual subjects, but also obviously in the primary visual cortex with classification accuracy rates as high as 93.7%.

Understanding the events of neural timing may provide RGB color decoding clues in vision (Martinovic, Mordal & Wuerger, 2011). The continuous of retinal images needs to be face by the visual system, each of these is related to keeping hundreds of milliseconds of neural activity (Marti & Dehaene, 2017). The system of visual needs a timeline to decode this information (when does it start? When does it end?). The time course of decoding RGB color EEG shows peaks relevant to stimulus start and stop. This enables events and times in a continuous flow of visual information to be aligned.

Then, do the only dimension temporal features for decoding EEG sufficiently suggest the neural representation in RGB color? Thus, it is complemented by cerebral cortex position. Compare with time, space position is a more outstanding and continued dimension. The current results supply the two dimensions with a neural correlation of possible. The current results are possibly open that there may be a third dimension that can connect these pieces of information.

Although it is easy to present the conventional spatial position of the EEG, which describe power for correspondence on channels, these dimensions lack more validation. Classification decoding builds associations between these dimensions, suggesting the neural mechanisms by which the brain encodes RGB color information.

Power and time dimensions provide the features of single channels and the relative relationship between channels. The design of the experiments was mainly purpose the evaluate hypothesis that the robustness of the single-channel features can be enhanced when using classification decoding as the third dimension. It has been examined in past physiological research work the features with time and power; in this work it is mainly tested the machine learning classifier, through time dimension into EEG raw data, training and testing data, and obtains independent classification decoding results on each channel. The results show that there are differences in classification accuracy between channels. Channel classification accuracy was significantly higher in the primary visual cortex than in other cortexes. At the same time, these channels are the same as the channels containing more power. These results were consistent with our prediction: both the decoding accuracy of the classification and the peak of the electrophysiological power are incremental on the same channel. Both of the results of the incremental distribution on cerebral cortex channels are consistent. We believe that on the one hand, the classification and decoding results obtained in machine learning reflect the neural representation of RGB colors, and on the other hand, this result is also a new dimension to support multivariate analysis of EEG data.

Between the classification decoding and the power distribution, the regarding knowledge still have a large gaps. We used the EEG high comparatively time determination and direct access to space association of them through the channel position. We used channel localization to estimate the subjects’ EEG signal sources. A method for concluding independent tests from classification decoding experiments was obtained from the results. For example, in the primary visual cortex, the strongest stimuli responses are on Oz, O1, O2, POz, PO3, PO4 channels (Wolffff et al., 2017); this condition has been described in previous research related to SSVEP. Between areas of the primal visual cortex, in response to the experiments of visual color stimulis, the EEG signal showed the great density of current source was present in the area assigned to the information of color. The difference between these stimuli just only in RGB color but not in other graphics, brightness, *etc*. Thus, the present finding supports the idea that the primary visual cortex has perceptions of pre-existing differential sensitivity to color information (Conway, 2018). In further work, we intend to use better neural network classifiers combined with Bayesian source localization analysis to explore the space position of intracranial processing of visual color information.

In summary, the signal resolution of spatial in EEG is lower than in MEG, and the techniques of EEG are more easily accessible in the lab over the world (Lopes da Silva, 2013). Despite Jasper E. Hajonides also studied visual color decoding using EEG signal data, his work was still based on the traditional semi-task-relevant classification. This research results on EEG visual color decoding has been vaguely characterized qualitatively, but it has not been quantitatively studied (Hajonides et al., 2021). The current work used EEG data multivariate analyses to reveal the RGB color information independent representations quantitatively. The work reveals multidimensional neural signatures of RGB color information, which may reflect the RGB color takes part in many calculations of visual.

**Figure 15:**
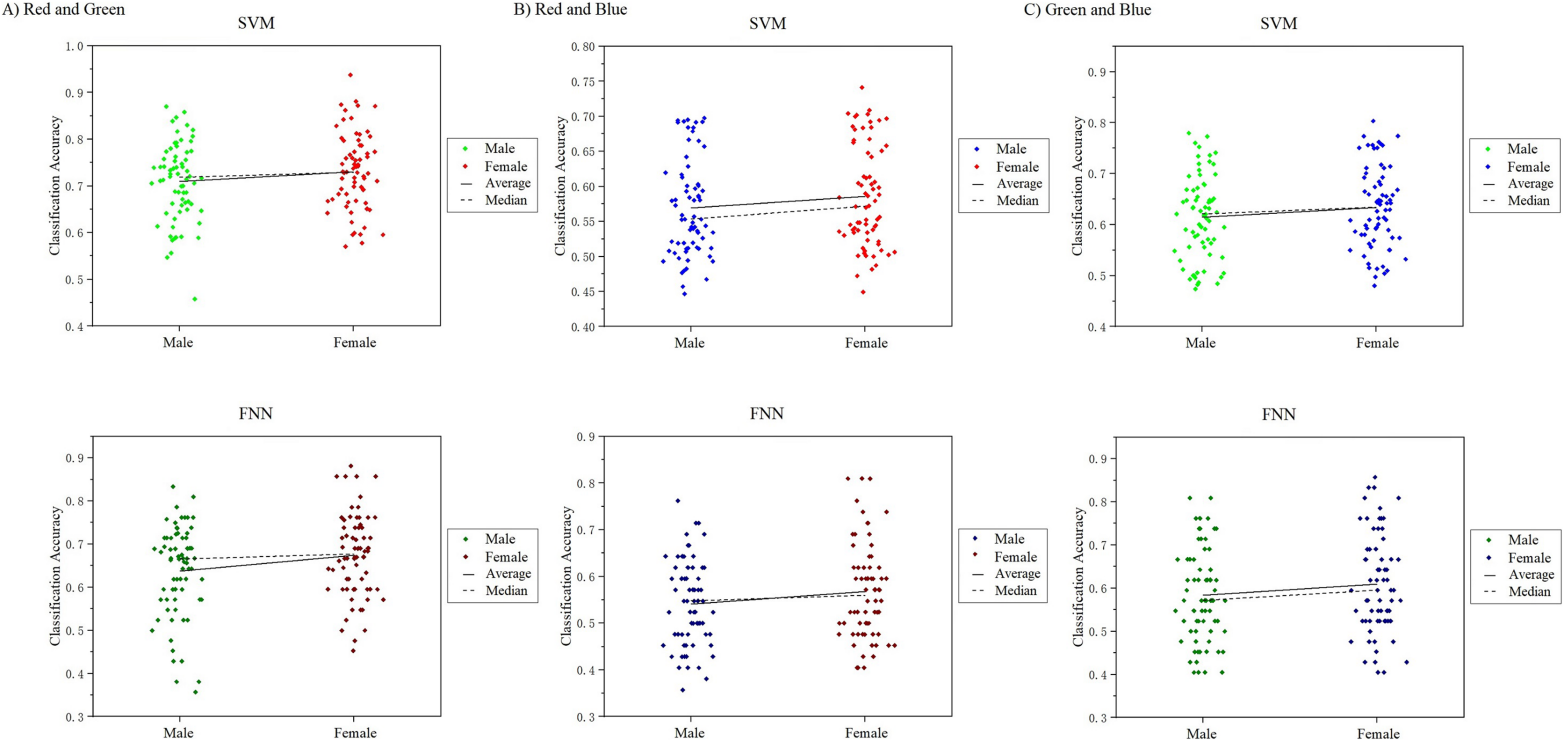
Comparison of classification results of male and female participants.

In this work, we noticed that the sample had an equal number of males and females. A good point during the course of the study was for us to analyze the visual color EEG of males and females to discuss whether there were significant differences between the sexes. We analyzed and compared the 10-channel classification decoding results of male subjects and female subjects on the primary visual cortex, and found that the decoding results of different genders for the observed color responses were basically similar (Fig. 15). In the RG group, the difference in mean and median results obtained in SVM and FNN results between males and females was 1.11%, 1.11%, 2.12%, 3.61%. In the RB group: 1.86%, 1.91%, 1.62%, 2.61%, in the GB group: 1.4%, 2.38%, 1.95%, 2.55%, the maximum difference is not more than 3% (Table S5). However, since our experiment is a small sample of data, it may not be able to fully reflect the results of the entire large group to a certain extent, which can be further explored and studied in future research.

## Conclusions

Previous scientific studies have explored various EEG information that exists in the primary visual cortex. However, there is still no clear conclusion where the brain region is sensitive to RGB which constitutes the basic elements of color. Therefore, it is important to investigate the neural representation and generation mechanism of visual RGB color sensing. Recent studies examines the color information sensing visual EEG responses. These EEG responses were further explored in temporal and spatial dimensions. The following conclusions are drawn from the results of experimental: (1) task-irrelevant has a new understanding of the EEG decoding of visual color information from a physiological point of view, breaking away from the traditional process of human perception of color through the scope of psychology; (2) task-irrelevant makes it possible to decode the visual color EEG independently, getting rid of the impure results (the EEG information of the data is polluted and the noise generated by other information) due to the need to fuse other information to participate in the traditional joint decoding; (3) machine learning provides another new dimension for traditional EEG decoding methods, and achieves high accuracy in temporal resolution; (4) using the decoding results of machine learning as leads, channel space distribution features (Pz, POz, Oz) containing color information were found based on visual EEG features, and the space features were quantified.

We arrived at the last four conclusions as follows: First, we started from the source of the research and carried out a new redesign of the experimental paradigm of the previous research. We will directly remove the various methods of psychology and decision science involved in the intervention from the experimental paradigm. The purpose of this design is to enable the subjects to receive only color stimuli and generate corresponding EEG perception signals. Although the generated signal will appear weaker compared to traditional interventional methods, this ensures that what we obtain is pure visual color EEG perception information. Secondly, when it is determined that what we obtain through the new paradigm design is the visual color EEG signal, compared with the traditional mixed signal, in the subsequent signal processing, not only the trouble of signal stripping is omitted, but also the loss of effective information is avoided. On the basis of the theory based on ERP and P300, by comparing the paradigm of SSVEP, it is feasible to introduce purely visual color stimulation as the main purpose of our experiment. In the subsequent research results, we also found that the visual color stimulus does contain the characteristics of ERP and P300 in the time dimension. Based on these features, we introduced a machine learning method to decode through temporal resolution. The findings suggest that it is feasible to use machine learning to decode EEG signals. Although some results are not ideal, most of the color information is still decoded effectively and fairly well. The perceptual areas of human vision have been identified in previous neurobiological studies and have been confirmed by many studies such as SSVEP. In this study, the results we obtained after decoding by machine learning are consistent with previous studies. Machine learning decoded EEG signals well in the primary visual cortex and achieved better classification results. The results of this study indicate that the EEG spatial information features of human visual color perception also exist in the primary visual cortex. However, anatomically elaborating primary visual cortex areas is a large area. Although it has the same characteristics in this area, it cannot be further specified to determine the detailed position of color perception. Therefore, our findings find another feature on top of the existing one. First, we were able to determine that the signal for color perception was generated in the same region as the signal for conventional visual perception. Based on this correct conclusion, we further analyzed the obtained decoding results and found that the visual nerve cells in Pz, POz, Oz channels are abnormally excited, and the perceptual signals generated after visual color stimulation are very strong. The result of this activity information is that strongly abnormal EEG signals are hattured by machine learning during the decoding process. Therefore, the four conclusions we obtained in the previous section were all obtained after a certain analysis based on the existing research theoretical basis and results, after introducing a new color mechanism and passing through a series of experiments.

In the current study, we used SVM and FNN as the classification decoder. However, the choice of classifiers is not limited to SVM and FNN, other classification models may also be applicable after modification. Through this work, it can be found that the currently used two classifiers perform well. To reveal the color decoding properties more important, it is necessary to use different performance classifiers in further research and extend the research to dynamic colors. However, this has the potential to provide a more full and accurate neural representation and generation mechanism decoding of visual color EEG positions for research. We encourage colleagues to try to use different classifiers to study the data. At the same time, we will continue to explore other methods in further research.

## Supplemental Information

10.7717/peerj-cs.1376/supp-1 Supplemental Information 1 Visual color stimulus adjustment and data preprocessing.

10.7717/peerj-cs.1376/supp-2 Supplemental Information 2 Environment and Error Adjustment and EEG data processing and analysis.

10.7717/peerj-cs.1376/supp-3 Supplemental Information 3 Parameters Description.

10.7717/peerj-cs.1376/supp-4 Supplemental Information 4 ROC analysis.

10.7717/peerj-cs.1376/supp-5 Supplemental Information 5 The significant difference between male and female.

## Additional Information and Declarations

### Data Availability

The following information was supplied regarding data availability: The data is available at the Science Data Bank: Wu Yi Jia. Decoding of the neural representation of the visual RGB colour model[DS/OL]. Science Data Bank, 2022[2023-03-02]. https://doi.org/10.57760/sciencedb.01763.
