# Supplementary material for: Decoding of the neural representation of the visual RGB color model
Source: PeerJ Comput Sci. 2023 May 11;9:e1376. doi: 10.7717/peerj-cs.1376 (PMC10280385; doi:10.7717/peerj-cs.1376)
Supplement: Supplemental Information 1 [file peerj-cs-09-1376-s001.docx]

| **Color adjustment** | | | | | | | | | | | | | |
| --- | --- | --- | --- | --- | --- | --- | --- | --- | --- | --- | --- | --- | --- |
| Color | | | | | RGB | | | | | HSL | | HEX | |
|  |  |  |  |  | R | | G | B | | Angle | | Code | |
| Pure | | Red | | | 255 | | 0 | 0 | | 0° | | #ff0000 | |
|  |  | Green | | | 0 | | 255 | 0 | | 120° | | #00ff00 | |
|  |  | Blue | | | 0 | | 0 | 255 | | 240° | | #0000ff | |
| **Data preprocessing** | | | | | | | | | | | | | |
| **FIR (Hz)** | | | **Sampling rate (Hz)** | | | **Eye movement artefacts** | | | **Baseline (ms)** | | **Record (ms)** | | |
| Raw | New | | Raw | New | | Remove | | | Start | | Start | | End |
| > 50 | < 30 | | 1000 | 200 | | Empty epoch | | | −200 | | 0 | | 1000 |
