# Supplementary material for: Decoding of the neural representation of the visual RGB color model
Source: PeerJ Comput Sci. 2023 May 11;9:e1376. doi: 10.7717/peerj-cs.1376 (PMC10280385; doi:10.7717/peerj-cs.1376)
Supplement: Supplemental Information 2 [file peerj-cs-09-1376-s002.docx]

| **Environment control** | | | | | | | | | | | | |
| --- | --- | --- | --- | --- | --- | --- | --- | --- | --- | --- | --- | --- |
|  | | | **Distance (cm)** | | | | | | **Channel** | | | |
| Location | | | View | | | Shelf | | | Fpz - O2 | | ECG - Veol | |
| Value | | | 36 | | | 15 | | | ＜ 3 kΩ | | × | |
| **Data analysis** | | | | | | | | | | | | |
| **Epoch** | **Red and Green** | | | **Red and Blue** | | | **Green and Blue** | | | **Cross-validation** | | |
| 3 trials | Red | Green | | Red | Blue | | Green | Blue | | Training (80%) | | Test (20%) |
|  | 105 | 105 | | 105 | 105 | | 105 | 105 | | 168 | | 42 |
| All | 210 | | | 210 | | | 210 | | | 210 | | |
| 30 times |  | | | | | | | | | 5040 | | 1260 |
