# Supplementary material for: Decoding of the neural representation of the visual RGB color model
Source: PeerJ Comput Sci. 2023 May 11;9:e1376. doi: 10.7717/peerj-cs.1376 (PMC10280385; doi:10.7717/peerj-cs.1376)
Supplement: Supplemental Information 3 [file peerj-cs-09-1376-s003.docx]

| **Property Name** | **Description** |
| --- | --- |
| Parameters | Structure containing the following fields:  LinearCoef: m-by-1 vector L.  OutputOffset: Scalar d. |
