# Supplementary material for: Decoding of the neural representation of the visual RGB color model
Source: PeerJ Comput Sci. 2023 May 11;9:e1376. doi: 10.7717/peerj-cs.1376 (PMC10280385; doi:10.7717/peerj-cs.1376)
Supplement: Supplemental Information 4 [file peerj-cs-09-1376-s004.docx]

| **Team** | **Model** | **AUC** | **Std.Error** | **Asymptotic Prob** | **95% LCL** | **95% UCL** |
| --- | --- | --- | --- | --- | --- | --- |
| RG | SVM | 0.893 | 0.015 | 9.4718259394056E-49 | 0.864 | 0.922 |
|  | FNN | 0.821 | 0.017 | 5.0234565185877E-33 | 0.788 | 0.853 |
| RB | SVM | 0.778 | 0.021 | 3.4138220301956E-25 | 0.737 | 0.819 |
|  | FNN | 0.643 | 0.023 | 9.581608398465E-8 | 0.598 | 0.687 |
| GB | SVM | 0.727 | 0.022 | 2.6916126193908E-17 | 0.684 | 0.769 |
|  | FNN | 0.685 | 0.021 | 4.535628688894E-12 | 0.643 | 0.727 |
