# Supplementary material for: Decoding of the neural representation of the visual RGB color model
Source: PeerJ Comput Sci. 2023 May 11;9:e1376. doi: 10.7717/peerj-cs.1376 (PMC10280385; doi:10.7717/peerj-cs.1376)
Supplement: Supplemental Information 5 [file peerj-cs-09-1376-s005.docx]

| **Sex** | **Stastic** | **Red and Green** | | **Red and Blue** | | **Green and Blue** | |
| --- | --- | --- | --- | --- | --- | --- | --- |
|  |  | **SVM** | **FNN** | **SVM** | **FNN** | **SVM** | **FNN** |
| Male | Average | 71.83% | 66.59% | 55.32% | 54.76% | 62.06% | 57.14% |
| Female |  | 72.94% | 67.70% | 57.18% | 55.95% | 63.46% | 59.52% |
| Male | Median | 70.92% | 63.80% | 56.93% | 54.12% | 61.42% | 58.37% |
| Female |  | 73.04% | 67.41% | 58.55% | 56.73% | 63.37% | 60.92% |
